# Consumo excessivo episódico de álcool no Nordeste do Brasil segundo raça/cor

**DOI:** 10.1590/0102-311XPT005524

**Published:** 2025-03-24

**Authors:** Renata da Silva Gomes, Amanda Cristina de Souza Andrade, Daiane Porto Nery, Daniele Sousa Portela, Vanessa Moraes Bezerra

**Affiliations:** 1 Instituto Multidisciplinar em Saúde, Universidade Federal da Bahia, Vitória da Conquista, Brasil.; 2 Universidade Federal de Mato Grosso, Cuiabá, Brasil.; 3 Programa de Pós-graduação em Saúde Pública, Universidade Federal de Minas Gerais, Belo Horizonte, Brasil.

**Keywords:** Bebedeira, Comportamentos de Risco à Saúde, População Negra, Estudos Epidemiológicos, Binge Drinking, Health Risk Behaviors, Black People, Epidemiologic Studies, Borrachera, Conductas de Riesgo para la Salud, Población Negra, Estudios Epidemiológicos

## Abstract

O objetivo deste estudo foi analisar os fatores associados ao consumo excessivo episódico de álcool na população da Região Nordeste do Brasil, segundo raça/cor. Realizou-se estudo transversal com dados da *Pesquisa Nacional de Saúde* de 2019, com indivíduos de 18 anos ou mais. O desfecho foi o consumo excessivo episódico de álcool medido pelo consumo de cinco ou mais doses de bebidas alcoólicas em uma única ocasião nos últimos 30 dias. Para avaliar a associação entre as variáveis sociodemográficas, de estilo de vida e de saúde/doença, foi utilizada a regressão de Poisson com variância robusta. As análises foram estratificadas por raça/cor. A prevalência de consumo excessivo episódico de álcool foi de 16,6% em brancos e 17,6% em negros, e para ambos os estratos, foi mais frequente entre: o sexo masculino; nos mais jovens; naqueles sem companheira(o); maior renda domiciliar *per capita*; presença de trabalho remunerado; ativos fisicamente; que faziam uso de tabaco e aqueles com hábitos alimentares inadequados. Maior escolaridade, não buscar os serviços de saúde e autoavaliação negativa da saúde se mantiveram associadas a consumo excessivo episódico de álcool somente no estrato de negros e na presença de três ou mais doenças crônicas e sobrepeso/obesidade entre os brancos. Características sociodemográficas, de estilo de vida e saúde/doença se associaram com o consumo excessivo episódico de álcool. Os fatores de saúde/doença associados ao consumo excessivo episódico de álcool foram diferentes entre os grupos de raça/cor. Os resultados reforçam a importância da implementação de ações intersetoriais, envolvendo órgãos de saúde e de regulação que visem à redução do consumo de bebidas alcóolicas e priorizem os grupos mais vulneráveis.

## Abstract (en)

This study aimed to analyze the factors associated with binge drinking in Northeastern Brazil according to race/skin color. A cross-sectional study was carried out with data from the 2019 *Brazilian National Health Survey* regarding individuals aged 18 years or older. The binge drinking outcome was measured by the consumption of five or more doses of alcoholic beverages on a single occasion in the previous 30 days. A Poisson regression with robust variance was used to evaluate the association between sociodemographic, lifestyle, and health/disease variables. The analyses were stratified by race/skin color. The prevalence of binge drinking equaled 16.6% in white people and 17.6% in black ones, occurring more often, for both strata, in men, younger people, those without a partner, those with higher per capita household income, those under paid work, physically active individuals, smokers, and those with inadequate eating habits. Higher schooling, seeking no health services, and negative self-perceived health remained associated with binge drinking only in black individuals and with three or more chronic diseases and overweight/obesity in white ones. Sociodemographic, lifestyle, and health/disease characteristics were associated with binge drinking. Health/disease factors associated with binge drinking differed between race/skin color groups. Results reinforce the importance of implementing intersectoral actions involving health and regulatory agencies to reduce the consumption of alcoholic beverages and prioritize the most vulnerable groups.

## Abstract (es)

El objetivo de este estudio fue analizar los factores asociados al consumo excesivo episódico de alcohol en la población de la Región Nordeste de Brasil según raza/color. Se realizó un estudio transversal con datos de la *Encuesta Nacional de Salud* de 2019, con personas de 18 años o más. El resultado fue consumo excesivo episódico de alcohol medido por el consumo de cinco o más dosis de bebidas alcohólicas en una sola ocasión en los últimos 30 días. Para evaluar la asociación entre las variables sociodemográficas, de estilo de vida y de salud/enfermedad se utilizó la regresión de Poisson con varianza robusta. Los análisis se estratificaron por raza/color. La prevalencia de consumo excesivo episódico de alcohol fue del 16,6% en blancos y del 17,6% en negros, y en ambos estratos fue más frecuente entre: hombres; en los más jóvenes; en aquellos sin pareja; con mayor ingreso familiar per cápita; con trabajo remunerado; físicamente activos; que usaban tabaco y aquellos con malos hábitos alimentarios. La educación superior, la no búsqueda de servicios de salud y la autoevaluación negativa de la salud permanecieron asociadas con el consumo excesivo episódico de alcohol solo en los negros; y la presencia de tres o más enfermedades crónicas y sobrepeso/obesidad entre los blancos. Las características sociodemográficas, de estilo de vida y de salud/enfermedad se asociaron con el consumo excesivo episódico de alcohol. Los factores de salud/enfermedad asociados al consumo excesivo episódico de alcohol fueron distintos entre los grupos de raza/color. Los resultados refuerzan la importancia de implementar acciones intersectoriales, involucrando a los centros de salud y regulatorios, para reducir el consumo de bebidas alcohólicas y priorizar a los grupos más vulnerables.

## Introdução

O consumo de bebidas alcoólicas é um comportamento cada vez mais presente no cotidiano das pessoas, sendo impulsionado por questões socioculturais e políticas como o reforço de tradições culturais que normalizam o consumo, a aplicação de impostos baixos, a liberalização das leis de venda e consumo, a falta de regulamentação rigorosa no marketing, a influência de grupos de interesse da indústria alcoólica e a carência de campanhas educativas sobre os riscos do consumo ^1^ ^,^ ^2^ ^,^ ^3^.

Por ser uma droga lícita, o acesso é mais facilitado e os malefícios provenientes do consumo atingem um número maior de pessoas ^4^. O consumo abusivo de álcool pode gerar consequências prejudiciais para a saúde e para a sociedade, como dependência, problemas mentais e hepáticos, neoplasias, entre outros. Além disso, é fator de riscos para violência, acidentes de trânsito e de trabalho ^5^.

Dentro dos diversos padrões de consumo de bebidas alcoólicas descritos na literatura, o consumo excessivo episódico de álcool é um dos mais utilizados para classificar os bebedores. Também conhecido como beber pesado episódico ou *binge drinking*, ele é caracterizado pela ingestão de 60g de álcool puro, equivalente a cerca de cinco doses de bebidas alcoólicas em uma única ocasião nos últimos 30 dias, tanto para homens quanto para mulheres ^4^ ^,^ ^6^.

Entre os determinantes do consumo excessivo episódico de álcool, destacam-se os socioculturais, nos quais o estímulo ao consumo do álcool está enraizado em diversas culturas, associado a celebrações, socializações e outros eventos sociais ^5^ ^,^ ^6^. Além disso, determinantes socioeconômicos, como renda e escolaridade, desempenham um papel crucial na definição de hábitos e comportamentos, incluindo o consumo excessivo de bebidas alcoólicas ^7^ ^,^ ^8^.

Outro fator que apresenta relação com consumo de álcool é a etnia e/ou raça/cor da pele. Considerada um determinante social no processo de saúde e doença, esse indicador impacta diretamente sobre outros determinantes sociais, além dos culturais, econômicos e de saúde dos indivíduos ^9^. Esses fatores não apenas contribuem para hábitos inadequados, como o consumo excessivo episódico de álcool, mas também podem acentuar as disparidades em saúde ^9^.

Atualmente, a presença do componente raça/cor nos estudos epidemiológicos tem ganhado relevância. Estudos brasileiros revelam que isso também está associado à maior prevalência do consumo excessivo episódico de álcool, pois aqueles de raça/cor da pele preta ou parda (população negra) estão mais propensos a praticá-lo ^10^ ^,^ ^11^. Esse fenômeno pode ser considerado uma consequência do sinergismo das desigualdades existentes no Brasil e pela combinação de fatores de riscos presentes em grande parte da população negra ^10^ ^,^ ^12^.

Entre as regiões brasileiras, a Nordeste é majoritariamente composta por indivíduos que se autodeclararam de cor da pele preta ou parda ^13^. Essa população é denominada como negra, e é conhecida por vivenciar historicamente condições sociais e de saúde desfavoráveis ^10^ ^,^ ^11^. Grande parte dessa população possui condições de vida mais precárias, observadas por indicadores de baixa escolaridade e renda e piores condições de saúde, quando comparado aos brancos ^9^ ^,^ ^10^ ^,^ ^11^. Sabe-se que condições socioeconômicas desfavoráveis podem influenciar diretamente na adoção de estilos de vida não saudáveis como o consumo excessivo episódico de álcool ^7^ ^,^ ^8^, tornando a população negra um grupo de risco.

Estudos recentes evidenciam um aumento significativo no consumo de bebidas alcoólicas na população brasileira ^5^ ^,^ ^10^ ^,^ ^11^. No entanto, há uma lacuna de pesquisas abordando o consumo excessivo episódico de álcool, especificamente na população negra. Dessa forma, o objetivo deste estudo foi analisar os fatores sociodemográficos (sexo, idade, renda domiciliar *per capita* e trabalho remunerado), de estilo de vida (tabagismo atual, atividade física e hábitos alimentares inadequados) e saúde/doença (índice de massa corporal [IMC], busca ao serviço de saúde nos últimos 15 dias, autoavaliação de saúde e presença de doenças crônicas não transmissíveis [DCNT]) associados ao consumo excessivo episódico de álcool em adultos da Região Nordeste do Brasil, estratificado por raça/cor.

## Métodos

Estudo transversal com dados da segunda edição da *Pesquisa Nacional de Saúde* (PNS 2019) ^14^. A amostra foi por conglomerado em três estágios: setores censitários; domicílios; um indivíduo com 15 anos ou mais, escolhido de forma aleatória, dentre os moradores da residência. O detalhamento do processo amostral pode ser consultado em Stopa et al. ^15^.

A PNS 2019 selecionou 94.114 domicílios e 88.531 indivíduos de 18 anos ou mais responderam ao questionário individual. Desses respondentes, 30.702 eram da Região Nordeste.

A variável dependente deste estudo foi o consumo excessivo episódico de álcool, medido pela ingestão de cinco ou mais doses de bebidas alcóolicas para homens ou mulheres em uma mesma ocasião nos últimos 30 dias ^6^. Considerando duas questões realizadas tanto para homens quanto para mulheres: “Com que frequência o(a) Sr(a) costuma consumir alguma bebida alcoólica? (não bebo nunca; menos de uma vez por mês; uma vez ou mais por mês)”, para aqueles que afirmaram alguma frequência no consumo de álcool, foi perguntado: “Nos últimos trinta dias, o(a) Sr(a) chegou a consumir cinco ou mais doses de bebidas alcoólica em uma única ocasião? (sim; não)” (uma dose de bebida alcoólica equivale a uma lata de cerveja, uma taça de vinho ou uma dose de cachaça, uísque ou qualquer outra bebida alcoólica destilada)” ^4^.

A análise foi estratificada pela variável raça/cor que foi dicotomizada em brancos (categoria branca) e negros (categorias parda e preta). Foram excluídos da análise os indivíduos que se autodeclararam de cor amarela (n = 1.698) e indígenas (n = 2.064).

**Figura 1:**
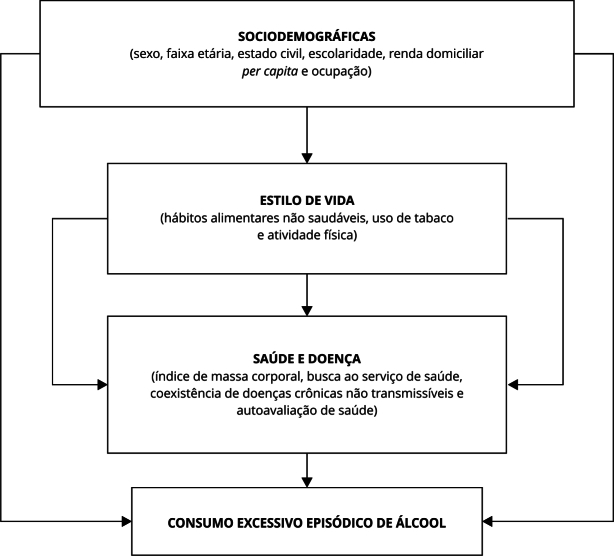
Modelo operacional para o conhecimento do consumo excessivo episódico de álcool.

As variáveis independentes foram organizadas em blocos, conforme estudos prévios ^7^ ^,^ ^10^ ^,^ ^16^: sociodemográficos, estilo de vida e saúde/doença (Figura 1). Para o bloco sociodemográfico, considerou-se as variáveis: sexo (masculino e feminino); idade (18-34; 35-59, 60 anos ou mais); estado civil (viver sem companheira/o englobando solteiros, divorciados e viúvos e viver com companheiro/a que incluiu os casados); escolaridade (sem instrução, Ensino Fundamental incompleto/completo, Ensino Médio incompleto/completo, Ensino Superior incompleto/completo); renda domiciliar *per capita* (até 1/2, > 1/2 a 1, > 1 a 2, > 2 salários mínimos) e trabalho remunerado (sim e não).

No bloco estilo de vida foram consideradas as variáveis tabagismo atual, atividade física e hábitos alimentares inadequados. O tabagismo atual foi avaliado a partir da pergunta: “Atualmente, o(a) Sr(a) fuma algum produto do tabaco?”. Foram classificados como fumantes atuais aqueles que responderam “sim, diariamente” ou “sim, menos que diariamente”.

A atividade física global foi mensurada através da multiplicação da frequência semanal (em dias) pela duração (em minutos) da prática de atividade física moderada e vigorosa em atividades de lazer, no trabalho, em atividades domésticas e no deslocamento. O tempo dispendido nas atividade física vigorosas foi multiplicado por dois. Aqueles que praticavam 150 minutos ou mais de atividade física por semana foram classificados como ativos ^17^.

Para a construção da variável “hábitos alimentares inadequados”, considerou-se o somatório das variáveis: consumo irregular de legumes ou verduras menor que cinco vezes por semana e de frutas menor que cinco vezes por semana; o consumo regular de refrigerante maior que cinco vezes por semana; e a substituição de refeições principais por lanches, sanduíches, salgados ou pizzas um dia ou mais na semana ^18^. A variável foi dicotomizada em zero hábito inadequado, um hábito inadequado, dois hábitos inadequados, três ou mais hábitos inadequados ^19^.

O bloco saúde/doença foi composto pelas variáveis: IMC, calculado a partir de peso e altura autorreferidos e classificado em “baixo peso/eutrofia” (< 18,5kg/m^2^ - baixo peso; ≥ 18,5kg/m^2^ e < 25kg/m^2^ - eutrofia) e “sobrepeso/obesidade” (< 30kg/m^2^ - sobrepeso; ≥ 30kg/m^2^ - obesidade) ^20^; busca ao serviço de saúde nos últimos 15 dias (sim; não); autoavaliação de saúde (muito boa/boa e regular/ruim/muito ruim); e presença de DCNT categorizada em nenhuma, uma, duas, três ou mais DCNT, de acordo com os dados autorreferidos de diagnósticos de hipertensão arterial, diabetes, colesterol alto, doença cardíaca, acidente vascular cerebral, asma, artrite, distúrbios osteomusculares relacionados ao trabalho, depressão, outra doença mental, doença pulmonar obstrutiva crônica, câncer, insuficiência renal crônica, outra doença crônica física ou mental.

A análise de dados foi realizada com o software Stata versão 15 (https://www.stata.com) e utilizado o módulo *survey*, que considera o desenho complexo da amostra e peso amostral. Foi calculada a prevalência de consumo excessivo episódico de álcool e seus respectivos intervalos de 95% de confiança (IC95%) e a frequência relativa para as variáveis sociodemográficas, de estilo de vida e de saúde/doença. Para a comparação das variáveis sociodemográficas, de estilo de vida e saúde/doença entre os estratos de raça/cor, foi utilizado o teste qui-quadrado de Pearson.

Foram realizadas análises bivariadas e múltiplas com estimativas de razões de prevalência (RP), cálculo dos respectivos valores de p e IC95% por meio de regressão de Poisson com variância robusta. As variáveis selecionadas para análise múltipla foram aquelas que apresentaram p < 0,20 na análise bivariada. Adotou-se entrada hierárquica das variáveis em blocos (Figura 1), obedecendo a seguinte ordem: sociodemográficas, estilo de vida e de saúde/doença. As variáveis de cada bloco e dos blocos hierarquicamente superiores foram ajustadas pelas variáveis do bloco seguinte. Todas as análises foram estratificadas por raça/cor. Foi utilizado critério de Akaike (AIC) para comparar os ajustes dos modelos e foi adotado um nível de significância de 5%.

A PNS teve aprovação da Comissão Nacional de Ética em Pesquisa do Conselho Nacional de Saúde (parecer nº 3.529.376).

## Resultados

Do total de entrevistados na Região Nordeste, 24,8% autodeclararam como brancos e 75,2% foram classificados como negros (junção de pretos e pardos). A prevalência do consumo excessivo episódico de álcool na população total foi de 17,3% (IC95%: 16,6-18,1), sendo 16,6% (IC95%: 15,3-17,9) entre os brancos e 17,6% (IC95%: 16,8-18,5) entre os negros, sem diferença estatística.

Diferenças significativas foram observadas entre os estratos de negros e brancos em relação a faixa etária, estado civil, escolaridade, renda domiciliar *per capita*, atividade física, uso de tabaco, coexistência de hábitos alimentares não saudáveis, presença de DCNT e autoavaliação de saúde (Tabela 1).

**Tabela 1:** Características da população de 18 anos ou mais, segundo estrato de raça/cor. Região Nordeste, Brasil, *Pesquisa Nacional de Saúde* 2019.

Variáveis	Total		Brancos		Negros		Valor de p *
	%	IC95%	%	IC95%	%	IC95%	
Consumo excessivo episódico de álcool							0,201
Não	82,7	81,9-83,4	83,4	82,1-84,7	82,4	81,5-83,2	
Sim	17,3	16,6-18,1	16,6	15,3-17,9	17,6	16,7-18,5	
Sociodemográficas							
Sexo							0,237
Masculino	46,5	45,5-47,4	45,6	43,9-47,3	46,7	45,6-47,9	
Feminino	53,6	52,6-54,5	54,4	52,7-56,1	53,3	52,1-54,4	
Faixa etária (anos)							< 0,001
18-34	33,6	32,7-34,5	31,4	29,8-33,1	34,3	33,2-35,4	
35-59	45,7	44,9-46,6	44,3	42,7-45,9	46,2	45,2-47,2	
60 ou mais	20,7	19,7-21,4	24,3	22,9-25-7	19,5	18,7-20,3	
Estado civil							< 0,001
Sem companheira/o	61,7	60,9-62,2	57,6	56,0-59,2	63,1	62,1-64,1	
Com companheira/o	38,3	37,4-39,1	42,4	40,8-44,0	36,9	35,9-37-9	
Escolaridade							< 0,001
Sem instrução	12,2	11,5-12,9	10,6	9,6-11,7	12,7	12,0-13,6	
Ensino Fundamental incompleto/completo	39,4	38,3-40,4	34,6	33,1-36,2	40,9	39,7-42,2	
Ensino Médio incompleto/completo	33,9	32,9-34,9	33,5	31,9-35,1	34,1	33,0-35,1	
Ensino Superior incompleto/completo	14,5	13,7-15,4	21,3	19,6-23,1	12,3	11,5-13,1	
Renda domiciliar *per capita* (salários mínimos)							< 0,001
Até 1/2	40,3	39,2-41,3	32,0	30,2-33,8	43,0	41,8-44,2	
> 1/2 a 1	33,0	32,1-34,0	31,5	29,8-33,4	33,5	32,4-34,6	
> 1 a 2	17,0	16,3-17,7	19,3	17,9-20,8	16,2	15,5-17,0	
> 2	9,7	9,0-10,4	17,2	15,6-18,9	7,2	6,7-7,8	
Trabalho remunerado							0,665
Não	51,0	50,0-51,9	50,7	49,0-52,3	51,1	50,0-52,2	
Sim	49,0	48,1-50,0	49,3	47,7-50,1	48,9	47,8-50,0	
Estilo de vida							
Atividade Física							< 0,001
Sim	60,9	59,9-61,8	57,9	56,3-59,5	61,9	60,7-63,0	
Não	39,1	38,2-40,1	42,1	40,5-43,8	38,2	37,1-39,3	
Uso de tabaco							< 0,001
Não	89,2	88,7-89,8	91,5	90,6-92,3	88,4	88,6-89,8	
Sim	10,8	10,2-11,4	8,5	7,7-9,4	11,6	10,2-11,4	
Hábitos alimentares não saudáveis							< 0,001
Nenhum	22,0	21,2-22,8	26,3	24,9-27,9	20,5	19,6-21,4	
1	31,8	31,0-32,6	33,0	31,5-34,6	31,4	30,5-32,3	
2	37,0	36,1-37,8	32,2	30,7-33,7	38,5	37,5-39,6	
3-4	9,3	8,8-9,9	8,5	7,6-9,5	9,6	9,0-10,2	
Saúde/Doença							
IMC							0,939
Baixo peso/Eutrofia	44,9	44,0-45,6	44,9	43,3-46,6	44,9	43,8-45,9	
Sobrepeso/Obesidade	55,1	54,2-56,0	55,1	53,4-56,7	55,1	54,1-56,1	
Busca dos serviços de saúde							0,192
Não	80,5	79,8-81,3	79,7	78,3-81,1	80,8	79,9-81,7	
Sim	19,5	18,7-20,3	20,3	18,9-21,7	19,2	18,3-20,1	
Presença de DCNT							0,030
Nenhuma	48,4	47,3-49,4	46,2	44,3-48,0	49,1	47,9-50,3	
1	25,6	24,8-26,5	26,3	24,8-27,9	25,4	24,5-26,4	
2	13,7	13,1-14,4	14,2	12,9-15,5	13,6	12,9-14,4	
3 ou mais	12,3	11,6-12,9	13,4	12,2-14,6	11,9	11,2-12,6	
Autoavaliação de saúde							< 0,001
Muito boa/Boa	56,7	55,7-57,7	59,9	58,0-61,7	55,7	54,5-56,8	
Regular/Muito ruim	43,3	42,3-44,3	40,2	38,4-42,0	44,3	43,2-45,5	

DCNT: doenças crônicas não transmissíveis; IC95%: intervalo de 95% de confiança; IMC: índice de massa corporal.

* Valor de p ≤ 0,05 (teste estatístico qui-quadrado).

Para ambos os estratos de raça/cor, as variáveis sexo masculino, faixas etárias mais jovens, viver sem companheiro, maiores níveis de escolaridade e renda domiciliar *per capita*, trabalho remunerado, ser ativo fisicamente, uso atual de tabaco e maior número de hábitos alimentares não saudáveis apresentaram associações positivas com o consumo excessivo episódico de álcool. Entre os brancos, foi observado efeito dose-resposta entre maiores níveis de escolaridade, maior renda e o consumo excessivo episódico de álcool. No que concerne ao bloco saúde/doença, o IMC não se associou significativamente com o consumo excessivo episódico de álcool em ambos os grupos. A busca aos serviços de saúde e coexistência de DCNT se associaram negativamente com o consumo excessivo episódico de álcool e autoavaliação de saúde se associou positivamente, em ambos os grupos (Tabela 2).

**Tabela 2:** Distribuição do consumo excessivo episódico de álcool na população de 18 anos ou mais, segundo estrato de raça/cor. Região Nordeste, Brasil, *Pesquisa Nacional de Saúde* 2019.

Variáveis	Brancos				Negros			
	%	RP	IC95%	Valor de p *	%	RP	IC95%	Valor de p *
Sociodemográficas								
Sexo								
Feminino	8,4	1,00			9,6	1,00		
Masculino	26,4	3,14	2,65-3,74	< 0,001	26,8	2,79	2,53-3,08	< 0,001
Faixa etária (anos)								
60 ou mais	5,6	1,00			5,5	1,00		
35-59	17,8	3,17	2,98-5,79	< 0,001	17,8	3,25	2,74-3,86	< 0,001
18-34	23,3	4,15	2,32-4,33	< 0,001	24,3	4,44	3,73-5,30	< 0,001
Estado civil								
Com companheira/o	13,0	1,00			11,6	1,00		
Sem companheira/o	19,2	1,49	1,27-1,74	< 0,001	21,1	1,82	1,64-2,01	< 0,001
Escolaridade								
Sem instrução	8,1	1,00			6,6	1,00		
Ensino Fundamental incompleto/completo	14,5	1,79	1,17-2,75	0,007	18,3	2,78	2,27-3,41	< 0,001
Ensino Médio incompleto/completo	19,0	2,35	1,55-3,58	< 0,001	20,4	3,10	2,57-3,74	< 0,001
Ensino Superior incompleto/completo	20,5	2,54	1,66-3,89	< 0,001	19,2	2,92	2,35-3,63	< 0,001
Renda domiciliar *per capita* (salários mínimos)								
Até 1/2	15,9	1,00			16,6	1,00		
> 1/2 a 1	14,4	0,91	0,73-1,30	0,387	16,8	1,01	0,90-1,14	0,832
> 1 a 2	16,6	1,05	0,83-1,32	0,703	19,7	1,19	1,04-1,35	0,007
> 2	21,9	1,38	1,11-1,72	0,003	22,9	1,38	1,18-1,60	< 0,001
Trabalho remunerado								
Não	9,2	1,00			11,2	1,00		
Sim	24,2	2,64	2,22-3,15	< 0,001	24,3	2,17	1,93-2,45	< 0,001
Estilo de vida								
Atividade física								
Não	10,4	1,00			11,6	1,00		
Sim	21,0	2,01	1,70-2,38	< 0,001	21,3	1,84	1,65-2,04	< 0,001
Uso de tabaco								
Não	15,0	1,00			15,8	1,00		
Sim	32,9	2,19	1,82-2,63	< 0,001	31,3	1,98	1,74-2,45	< 0,001
Hábitos alimentares não saudáveis								
Nenhum	11,4	1,00			13,0	1,00		
1	16,8	1,48	1,20-1,83	< 0,001	16,9	1,30	1,11-1,52	0,001
2	18,3	1,61	1,29-2,00	< 0,001	18,2	1,40	1,21-1,61	< 0,001
3-4	25,2	2,22	1,71-2,87	< 0,001	27,5	2,11	1,78-2,50	< 0,001
Saúde/Doença								
IMC								
Baixo peso/Eutrofia	15,4	1,00			17,9	1,00		
Sobrepeso/Obesidade	17,7	1,15	0,98-1,35	0,089	17,6	0,98	0,89-1,08	0,683
Busca dos serviços de saúde								
Sim	12,6	1,00			11,4	1,00		
Não	17,6	1,39	1,11-1,74	< 0,001	19,1	1,67	1,46-1,91	< 0,001
Presença de DCNT								
Nenhuma	19,8	1,00			21,0	1,00		
1	15,2	0,77	0,61-0,97	0,024	15,4	0,73	0,66-0,81	< 0,001
2	11,9	0,60	0,46-0,79	< 0,001	10,7	0,51	042-0,61	< 0,001
3 ou mais	5,5	0,28	0,19-0,40	< 0,001	7,6	0,36	0,30-0,45	< 0,001
Autoavaliação de saúde								
Muito boa/Boa	19,9	1,00			21,2	1,00		
Regular/Ruim/Muito ruim	11,6	0,58	0,49-0,69	< 0,001	13,0	0,61	0,55-0,68	< 0,001

DCNT: doenças crônicas não transmissíveis; IC95%: intervalo de 95% de confiança; IMC: índice de massa corporal; RP: razão de prevalência.

* Valor de p ≤ 0,05 (teste estatístico qui-quadrado).

Na análise multivariada para o estrato de brancos (Tabela 3), observou-se no modelo 1 associação positiva das variáveis sexo masculino, faixas etárias mais jovens, viver sem companheira/o, a maior categoria de renda e ter trabalho remunerado com consumo excessivo episódico de álcool. No modelo 2, ser ativo fisicamente, uso de tabaco e presença de um ou mais hábitos alimentares não saudáveis se associaram positivamente ao consumo excessivo episódico de álcool. No modelo 3 ajustado por todos os blocos de variáveis, o sobrepeso/obesidade se associou positivamente ao consumo excessivo episódico de álcool, já a coexistência de três a quatro DCNT se associaram negativamente com o desfecho.

**Tabela 3:** Modelo hierarquizado dos fatores associados ao consumo excessivo episódico de álcool na população branca. Região Nordeste, Brasil, *Pesquisa Nacional de Saúde* 2019.

Variáveis	Modelo 1		Modelo 2		Modelo 3	
	RP	IC95%	RP	IC95%	RP	IC95%
Sociodemográficas						
Sexo						
Feminino	1,00		1,00		1,00	
Masculino	3,03	2,66-3,46	2,80	2,45-3,21	2,62	2,27-3,03
Faixa etária (anos)						
60 ou mais	1,00		1,00		1,00	
35-59	2,65	2,15-3,27	2,53	2,04-3,12	2,43	1,94-3,05
18-34	3,15	2,53-3,92	2,99	2,39-3,74	2,93	2,30-3,74
Estado civil						
Com companheira/o	1,00		1,00		1,00	
Sem companheira/o	1,48	1,30-1,67	1,38	1,22-1,57	1,36	1,19-1,56
Renda domiciliar *per capita* (salários mínimos)						
Até 1/2	1,00		1,00		1,00	
> 1/2 a 1	1,05	0,90-1,23	1,11	0,95-1,30	1,18	0,99-1,42
> 1 a 2	1,25	1,06-1,47	1,34	1,13-1,58	1,45	1,21-1,75
> 2	1,48	1,25-1,74	1,61	1,36-1,90	1,80	1,50-2,17
Trabalho remunerado						
Não	1,00		1,00		1,00	
Sim	1,58	1,39-1,84	1,51	1,31-1,74	1,45	1,24-1,70
Estilo de vida						
Atividade física						
Não			1,00		1,00	
Sim			1,37	1,20-1,56	1,45	1,26-1,67
Uso de tabaco						
Não			1,00		1,00	
Sim			1,92	1,66-2,24	2,04	1,72-2,43
Hábitos alimentares não saudáveis						
Nenhum			1,00		1,00	
1			1,13	0,96-1,33	1,13	0,95-1,34
2			1,16	0,98-1,37	1,18	0,99-1,40
3-4			1,27	1,03-1,58	1,23	0,97-1,56
Saúde/Doença						
IMC						
Baixo peso/Eutrofia					1,00	
Sobrepeso/Obesidade					1,28	1,12-1,46
Presença de DCNT						
Nenhuma					1,00	
1					0,91	0,78-1,06
2					0,96	0,78-1,18
3 ou mais					0,71	0,54-0,93
AIC	6.126,19		6.045,01		5.164,5	

AIC: critério de Akaike; DCNT: doenças crônicas não transmissíveis; IC95%: intervalo de 95% de confiança; IMC: índice de massa corporal; RP: razão de prevalência.

Nota: valor de p < 0,05; regressão de Poisson. Modelo 1: ajustado entre as variáveis sociodemográficas; modelo 2: ajustado entre as variáveis sociodemográficas e de estilo de vida; modelo 3: ajustado entre as variáveis sociodemográficas, estilo de vida e saúde/doença.

Na análise multivariada para o estrato de negros (Tabela 4), no modelo 1 se observou associação positiva das seguintes variáveis com o consumo excessivo episódico de álcool: sexo masculino; faixas etárias mais jovens; estado civil viver sem companheira/o; maiores níveis de escolaridade; renda domiciliar *per capita* maior que ½ salário mínimo e ter trabalho remunerado. No modelo 2 foi observada associação positiva daqueles fisicamente ativos, do uso atual de tabaco e presença de um ou mais hábitos alimentares inadequados com o desfecho. No modelo 3, observou-se associação positiva da “não buscar aos serviços de saúde” e uma associação negativa da autoavaliação de saúde regular/muito ruim com o consumo excessivo episódico de álcool (Tabela 4).

**Tabela 4:** Modelo hierarquizado dos fatores associados ao consumo excessivo episódico de álcool na população negra. Região Nordeste, Brasil, *Pesquisa Nacional de Saúde* 2019.

Variáveis	Modelo 1		Modelo 2		Modelo 3	
	RP	IC95%	RP	IC95%	RP	IC95%
Sociodemográficas						
Sexo						
Feminino	1,00		1,00		1,00	
Masculino	2,65	2,47-2,85	2,47	2,30-2,66	2,40	2,23-2,58
Faixa etária (anos)						
60 ou mais	1,00		1,00		1,00	
35-59	2,32	2,05-2,63	2,15	1,90-2,44	2,10	1,85-2,38
18-34	2,70	2,36-3,09	2,43	2,12-2,79	2,30	2,00-2,64
Estado civil						
Com companheira/o	1,00		1,00		1,00	
Sem companheira/o	1,62	1,51-1,74	1,52	1,41-1,64	1,51	1,41-1,63
Escolaridade						
Sem instrução	1,00		1,00		1,00	
Ensino Fundamental incompleto/completo	1,64	1,43-1,89	1,70	1,49-1,96	1,71	1,49-1,96
Ensino Médio incompleto/completo	1,60	1,39-1,86	1,77	1,53-2,05	1,76	1,51-2,04
Ensino Superior incompleto/completo	1,51	1,27-1,79	1,72	1,45-2,04	1,70	1,43-2,02
Renda domiciliar *per capita* (salários mínimos)						
Até 1/2	1,00		1,00		1,00	
> 1/2 a 1	1,11	1,02-1,20	1,10	1,02-1,19	1,11	1,02-1,20
> 1 a 2	1,23	1,39-1,86	1,23	1,12-1,36	1,24	1,13-1,36
> 2	1,47	1,27-1,79	1,47	1,30-1,67	1,46	1,29-1,66
Trabalho remunerado						
Não	1,00		1,00		1,00	
Sim	1,53	1,42-1,65	1,46	1,35-1,57	1,43	1,33-1,55
Estilo de vida						
Atividade física						
Não			1,00		1,00	
Sim			1,28	1,19-1,38	1,28	1,19-1,38
Uso de tabaco						
Não			1,00		1,00	
Sim			1,73	1,60-1,86	1,73	1,60-1,87
Hábitos alimentares não saudáveis						
Nenhum			1,00		1,00	
1			1,16	1,05-1,28	1,15	1,05-1,27
2			1,16	1,05-1,28	1,15	1,05-1,27
3-4			1,50	1,33-1,69	1,50	1,33-1,69
Saúde/Doença						
Busca dos serviços de saúde						
Sim					1,00	
Não					1,23	1,12-1,35
Autoavaliação de saúde						
Muito boa/Boa					1,00	
Regular/Ruim/Muito ruim					0,87	0,81-0,93
AIC	19.476,41		19.229,55		16.110,28	

AIC: critério de Akaike; DCNT: doenças crônicas não transmissíveis; IC95%: intervalo de 95% de confiança; IMC: índice de massa corporal; RP: razão de prevalência.

Nota: valor de p < 0,05; regressão de Poisson. Modelo 1: ajustado entre as variáveis sociodemográficas; modelo 2: ajustado entre as variáveis sociodemográficas e de estilo de vida; modelo 3: ajustado entre as variáveis sociodemográficas, estilo de vida e saúde/doença.

## Discussão

Neste estudo foi observada associação de características sociodemográficas, de estilo de vida e saúde/doença com o consumo excessivo episódico de álcool entre residentes da Região Nordeste do Brasil. Não foram observadas diferenças na prevalência do consumo excessivo episódico de álcool entre brancos e negros. Contudo, alguns fatores associados ao consumo excessivo episódico de álcool foram diferentes entre os estratos. As variáveis escolaridade, busca por serviço de saúde e autoavaliação de saúde se mantiveram associadas no modelo final apenas no estrato de negros, e a presença de doenças crônicas e sobrepeso/obesidade no estrato de brancos.

Estudos recentes indicam um aumento global no consumo de álcool ^4^ ^,^ ^16^. O Brasil apresenta prevalências de consumo (19,4%) superiores à média global (18,2%) ^4^. Neste estudo, a Região Nordeste apresentou uma prevalência de consumo excessivo episódico de álcool (17,3%) inferior à média nacional. Embora haja uma estabilização recente do consumo excessivo episódico de álcool na Região Nordeste, outras regiões demonstram uma redução desse comportamento de risco ^4^. Além disso, uma pesquisa conduzida com jovens adultos brasileiros mostrou que a Região Nordeste apresentou maiores prevalências de consumo excessivo episódico de álcool em comparação com outras regiões do país ^7^.

Neste estudo, não foram observadas diferenças significativas na prevalência de consumo excessivo episódico de álcool entre as populações de brancos e negros. No entanto, pesquisa com dados nacionais identificou uma maior propensão ao consumo excessivo episódico de álcool entre aqueles que se identificaram como preto ou pardo em comparação aos brancos ^10^. A combinação de fatores de risco, como baixos níveis de renda e escolaridade ^9^ ^,^ ^10^, amplifica os efeitos prejudiciais do álcool na população negra, justificando a demanda de um cuidado especial nesse grupo.

No presente trabalho, indivíduos do sexo masculino e aqueles mais jovens apresentaram maior prevalência do consumo excessivo episódico de álcool, independente da raça/cor. O consumo de álcool é reconhecido como um símbolo de masculinidade, em que experiências com a bebida são consideradas mais importantes para os homens. Além disso, aspectos fisiológicos como maior tolerância ao álcool em função da sua farmacocinética diferenciada ^10^, influências culturais, do contexto socioeconômico e o trabalho podem justificar o maior consumo excessivo episódico de álcool pela população masculina ^21^.

Prevalências elevadas do consumo excessivo episódico de álcool em adultos mais jovens foram identificadas em outros estudos ^5^ ^,^ ^22^. A falta de fiscalização no consumo de álcool, aliada ao estímulo da sociedade por meio de estratégias de marketing da indústria do álcool direcionadas a esse público, podem influenciar a adoção desse comportamento de risco ^3^. Mesmo que sexo e idade tenham sido associados em ambos os estratos estudados, os impactos negativos do consumo excessivo episódico de álcool podem ser mais acentuados na população masculina negra e nos jovens negros ^9^ ^,^ ^10^ ao considerar o contexto social em que estão inseridos. Esses grupos frequentemente enfrentam condições de vida mais desafiadoras em comparação aos brancos, e, nesse contexto, outros comportamentos de risco podem coexistir com o consumo excessivo episódico de álcool ^9^ ^,^ ^10^.

Indivíduos que relataram não ter companheira/o apresentaram maior prevalência de consumo excessivo episódico de álcool em ambos os estratos. De acordo com a literatura, solteiros apresentam essas maiores prevalências do consumo excessivo episódico de álcool, visto que podem frequentar mais eventos de socialização, como festas e reuniões, que apresentam maior disponibilidade de bebidas alcoólicas para consumo ^7^. Ademais, é reconhecido que, a união estável parece exercer um papel importante na manutenção de hábitos de vida saudáveis e no menor envolvimento com comportamentos de riscos ^23^.

Ter uma ocupação remunerada e possuir renda mais alta foram associados a maiores prevalências do consumo excessivo episódico de álcool, em brancos e negros. No entanto, níveis educacionais mais elevados mostraram uma associação positiva com consumo excessivo episódico de álcool apenas entre os negros. Estudos indicam uma relação direta entre o aumento da escolaridade e renda com o consumo de álcool ^7^ ^,^ ^8^. É reconhecido que, em geral, a população negra possui menor escolaridade em comparação aos brancos ^7^, conforme também observado nesta pesquisa. Contudo, as mudanças nos níveis educacionais entre os negros não apenas refletem em aspectos socioeconômicos ^11^ ^,^ ^24^, mas também na capacidade de adquirir mais conhecimento e cuidar da saúde ^25^. A alta renda e níveis educacionais mais elevados estão associados a uma maior ingestão de álcool, pois contribuem para uma vida social mais ativa, facilitando o consumo em ambientes sociais como confraternizações, lazer e reuniões de trabalho ^10^ ^,^ ^11^ ^,^ ^24^.

Em relação ao estilo de vida, a presença de hábitos alimentares não saudáveis, o uso atual de tabaco e a atividade física apresentaram associação positiva com o consumo excessivo episódico de álcool, em ambas as populações. Os hábitos alimentares inadequados estiveram presentes entre negros e brancos, sendo mais frequente na população negra. A combinação de bebidas alcoólicas e alimentos não saudáveis pelos bebedores pode justificar essa associação ^26^. Contudo, outros cenários podem influenciar na adoção de hábitos alimentares. Sabe-se que a escolha dos alimentos pode estar associada ao seu valor monetário, assim, indivíduos que vivem em contextos economicamente desfavorecidos, a exemplo da população negra, podem não ter opção de escolhas mais saudáveis e/ou condições de adquiri-lás ^27^.

Quanto à associação positiva observada do fumo com consumo excessivo episódico de álcool, estudos apontam que o consumo de uma substância pode desencadear a ingestão de outras, assim, o consumo de álcool e tabaco estão diretamente associados ^10^. Maior prevalência do tabagismo entre os negros, comparados aos brancos, foi observada no presente estudo e na população brasileira ^28^. As piores condições socioeconômicas e menor urbanização são considerados fatores de risco para o tabagismo no Brasil ^29^. Considerando que uma grande parcela dos negros brasileiros está inserida em um contexto de vulnerabilidade socioeconômica, esse contexto pode justificar a adoção de comportamentos de risco como os tabagismos e o consumo excessivo episódico de álcool nessa população ^28^ ^,^ ^29^.

Em relação à associação positiva da atividade física com o consumo excessivo episódico de álcool, sabe-se que o nível despendido de atividade física engloba diversos domínios como lazer, trabalho, atividades domésticas e deslocamento ^17^. Uma maior prevalência de consumo excessivo episódico de álcool entre os indivíduos ativos pode ser explicada pela participação frequente em eventos sociais que envolvem consumo de álcool após atividades físicas de lazer ^30^ e pelo uso do consumo excessivo episódico de álcool como forma de aliviar o estresse relacionado ao ambiente de trabalho ^21^. Porém, é importante considerar a contribuição dos demais domínios de atividade física no somatório da atividade física global. Grupos vulneráveis a exemplo da população do Nordeste e de negros estão mais propensos a realizarem atividades laborais não formais e/ou realizarem mais trabalhos braçais, podem também residir longe dos seus locais de trabalho e muitas vezes realizar o deslocamento a pé ou de bicicleta, situações que contribuem para uma maior atividade física no trabalho e no deslocamento ^12^. Atrelado a isso, tem-se o trabalho doméstico no Brasil, que é essencialmente feminino e corresponde a cerca de 16,8% dessa população total, sendo que desse contingente 61% são compostos por mulheres negras ^31^, situação que pode contribuir para uma maior atividade física no domínio doméstico nesse grupo e assim contribuir para uma maior atividade física global entre os negros.

A condição de sobrepeso/obesidade manteve-se significativamente associada ao consumo excessivo episódico de álcool somente no grupo de brancos. As bebidas alcóolicas são reconhecidas pela sua elevada densidade calórica e por serem pobres em nutrientes, além de frequentemente consumidas em conjunto com alimentos calóricos e pouco nutritivos, e por isso contribuem diretamente no ganho de peso, o que pode justificar a associação entre obesidade e consumo excessivo episódico de álcool ^24^.

Neste estudo, indivíduos classificados com três ou mais DCNTs apresentaram menor prevalência do evento no estrato de brancos. O consumo de bebidas alcoólicas está associado ao desenvolvimento de DCNT, assim, uma possível justificativa para esse achado é o fato de os indivíduos portadores de doenças crônicas como diabetes, doença renal crônica e hipertensão arterial frequentarem mais os serviços de saúde e receberem orientações sobre evitar o consumo de bebidas alcoólicas ^4^.

Somente no grupo dos negros foram observadas associações significativas entre a busca por serviços de saúde, a autoavaliação de saúde e o consumo excessivo episódico de álcool. Aqueles que afirmaram não buscar os serviços de saúde apresentaram maior prevalência do evento. Fatores inerentes ao contexto de vida da maioria da população negra refletem diretamente no acesso, conhecimento e cuidados em saúde. Sabe-se que essa população sofre maiores restrições no acesso a serviços de saúde, e estes, quando disponibilizados, são de pior qualidade e menor resolutividade ^25^. Esse cenário pode justificar a elevada prevalência do consumo excessivo episódico de álcool no grupo de negros que não buscam os serviços de saúde e reforça a importância da garantia efetiva do acesso aos serviços de saúde, em especial na população negra.

Em relação a autoavaliação de saúde, os indivíduos classificados como negros, que avaliaram sua saúde como regular/muito ruim, consumiam menos álcool. Existe uma associação entre autopercepção de saúde e DCNT, indivíduos que avaliaram sua saúde como regular/muito ruim possuíam em maior frequência de três ou mais DCNTs (dados não apresentados em tabela). Uma frequente associação entre a autoavaliação negativa do estado de saúde com a presença de morbidades crônicas tem sido encontrada na população geral ^32^. A autoavaliação do estado de saúde engloba aspectos multidimensionais e subjetivos dos indivíduos, a exemplo, tem-se maiores prevalências de autoavaliação da saúde como ruim encontradas na Região Nordeste ^32^, que apresenta como marcadores desigualdades na autoavaliação de saúde, menores níveis de renda domiciliar *per capita*, menor escolaridade, pertencer às classes sociais mais baixas e cor de pele parda e preta ^32^.

Quanto às limitações deste estudo, tem-se delineamento transversal do estudo, não sendo possível identificar a natureza temporal de algumas associações encontradas. A determinação do consumo excessivo episódico de álcool baseada na condição referida pelo entrevistado pode gerar um viés de informação, superestimando ou subestimando os resultados. Contudo, a fim de minimizar falhas, a PNS realiza padronização e treinamento dos entrevistadores para a realização das perguntas e/ou coleta dos dados da pesquisa, garantindo a qualidade dos mesmos ^15^.

Os achados desse estudo reafirmam o papel dos determinantes sociais no processo de saúde e doença, e a necessidade de políticas públicas e ações eficazes de enfrentamento desse relevante comportamento de risco, que desencadeia aumento na carga de doenças e mortalidades no cenário brasileiro, principalmente entre os negros. Características sociodemográficas, de estilo de vida e saúde/doença estiveram associados ao consumo excessivo episódico de álcool nesse grupo.

Os resultados reforçam a importância da implementação de ações intersetoriais, envolvendo órgãos de saúde e de regulação, que visem à redução do consumo de bebidas alcóolicas e priorizem os grupos mais vulneráveis.
